# Global crop-specific nitrogen fertilization dataset in 1961–2020

**DOI:** 10.1038/s41597-023-02526-z

**Published:** 2023-09-11

**Authors:** Wulahati Adalibieke, Xiaoqing Cui, Hongwei Cai, Liangzhi You, Feng Zhou

**Affiliations:** 1 Institute of Carbon Neutrality, Laboratory for Earth Surface Processes, College of Urban and Environmental Sciences, Peking University, Beijing, 100871 China; 2 International Food Policy Research Institute (IFPRI), Washington, DC20005 USA

**Keywords:** Agriculture, Geography, Element cycles

## Abstract

Nitrogen (N) is an important nutrient for crop growth. However, the overuse of N fertilizers has led to a series of devastating global environmental issues. Recent studies show that multiple datasets have been created for agricultural N fertilizer application with varied temporal or spatial resolutions, nevertheless, how to synchronize and use these datasets becomes problematic due to the inconsistent temporal coverages, spatial resolutions, and crop-specific allocations. Here we reconstructed a comprehensive dataset for crop-specific N fertilization at 5-arc-min resolution (~10 km by 10 km) during 1961–2020, including N application rate, types, and placements. The N fertilization data was segmented by 21 crop groups, 13 fertilizer types, and 2 fertilization placements. Comparison analysis showed that our dataset is aligned with previous estimates. Our spatiotemporal N fertilization dataset could be used for the land surface models to quantify the effects of agricultural N fertilization practices on food security, climate change, and environmental sustainability.

## Background & Summary

Nitrogen (N) is one of the most essential nutrients for crop production. Since the development of the Haber-Bosch process, the consumption of synthetic N fertilizers has increased exponentially to meet the food demand of growing human population^1,2^. However, excess N fertilizers are escaping into the environment, via a cascade of processes through atmospheric, aquatic, marine and terrestrial pools^3,4^. For example, agricultural N pollutants due to emitting, run-offs and leaching could result in severe air pollution (i.e., fine particle matter and aerosols)^5,6^, global climate change (i.e., ozone depletion in the stratosphere)^7,8^, eutrophication^9^ and compromised water quality in surface and groundwater aquatic ecosystems^10,11^, biodiversity loss^12^ as well as acidification in terrestrial ecosystems^13^. In order to assess the effect of N fertilization, which was defined as N application rate, types (including synthetic N fertilizers, manure and crop residues) and placement, on the environment at different scales (especially for a globally large scale), diverse datasets of agricultural N fertilization practices segmented by various temporal and spatial resolutions are urgently needed.

N fertilization has been considered as an important input in multiple models, e.g., the Organising Carbon and Hydrology In Dynamic Ecosystems model (ORCHIDE-Crop)^6^ and PKU-NRUNOFF model^14^ require crop-specific N inputs, the Soil & Water Assessment Tool (SWAT)^15^ and the Magnitude And Seasonality of Agricultural Emissions model for NH_3_ (MASAGE_NH3 models)^16^ require fertilizer types as input data besides. More importantly, the DeNitrification-DeComposition model (DNDC)^17,18^ and the flux upscaling model for N_2_O emissions^19^ simultaneously need crop-specific N inputs, fertilizer types, and application placement as input data. Generally, the country-level N fertilizer applied to agriculture could be obtained from the Food and Agriculture Organization of the United Nations (FAO)^20^ and the International Fertilizer Association (IFA)^21^, which have been widely used in global and national research. However, detailed geospatial distributions, which are usually required in many process-based models, are unavailable from these international statistics. Crop-specific N fertilizer application rate at a resolution of 5 arc-min was firstly generated by Mueller *et al*.^22^, whereas this study only focused on the rates for the year 2000. The related dataset was further expanded to a period of 1961–2013 at a relatively lower resolution of 0.5 degree × 0.5 degree and segmented by NH_4_^+^ and NO_3_^−^ forming N fertilizers^23^.Moreover, Wang *et al*.^24^ extended the temporal period of N application rate data into the year 1961–2014, but only into two categories (i.e., upland crops and paddy rice) through aggregating administrative-unit statistic data. Meanwhile, a 5-arc-min gridded global dataset pertaining to global manure N production and application in cropland during 1860–2015 was reconstructed by Zhang *et al*.^25^. Historic anthropogenic N inputs to terrestrial biosphere with 5 arc-min during 1960–2019 were also reconstructed, including annual rates of synthetic N fertilizer, manure application, and atmospheric N deposition on cropland, pasture, and rangeland^26^. It is noted that most current research and datasets mainly focus on N fertilization inputs instead of N fertilizer types and N fertilization placement. Moreover, those datasets did not show long-term and high-resolution patterns of crop-specific N fertilization (rate, type, placement) and were not at uniform spatial resolutions or with consistent allocation algorithms, making it impossible to use them together.

To provide input data for the land surface models for assessing the effects of agricultural N fertilization practices on food security, climate change and ecosystem health, we developed a new global crop-specific N fertilization dataset. This newly developed N fertilization dataset covers not only more than one single fertilization rate but also crop-specific N application rate, N fertilizer types, as well as N application placement at a spatial resolution of 5 arc-min in the past six decades. This dataset was developed based on multiple statistical datasets (e.g., the FAOSTAT^20^, the IFASTAT^21^, and the EUROSTAT^27^), empirical estimates as well as gridded dataset (e.g., gridded harvested area from the EARTHSTAT^28^, gridded N application for Wang *et al*.^24^ and Zhan *et al*.^29^ and gridded climate data from Climatic Research Unit (CRU)^30^). This dataset also combines previous datasets at different spatial and temporal scales to reconstruct the important components of N fertilization (i.e., rate, type and placement) by crop types. The uncertainties of crop-specific N application rate were estimated and further comparison between our new N fertilization dataset and existing N fertilization datasets were also analyzed. The limitations that users should be aware and the future research priorities to build a more accurate and comprehensive N fertilization dataset were also discussed in the “Usage Notes”.

## Methods

**Fig. 1:**
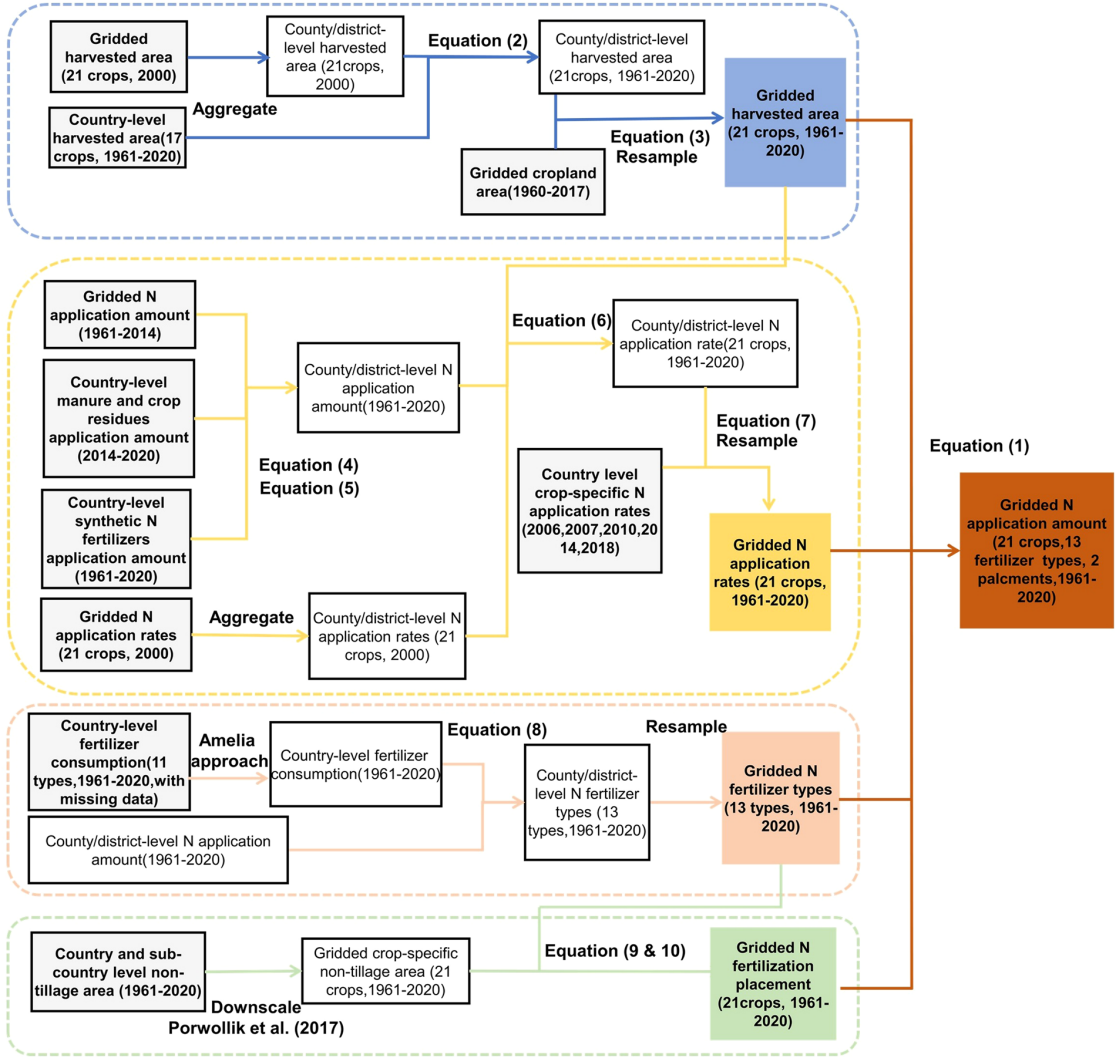
Workflow for developing the crop-specific N application dataset.

A comprehensive crop-specific N fertilization dataset was reconstructed across the global agricultural soils, including the N application applied to twenty-one crops groups with different fertilizer types and application methods. The N fertilization was defined as N application of synthetic N fertilizers, crop residues and manure to cropland in this study. The N application amount can be quantified as below:

1 $$\begin{array}{c}Namoun{t}_{cro,f,p,y,g}=Hare{a}_{cro,y,g}\times Nrat{e}_{cro,y,g}\times FerRati{o}_{f,y,g}\times Placemen{t}_{cro,f,p,y,g}\end{array}$$

where *Namount*_*cro,f,p,y,g*_ (kg N) is N application amount of fertilizer type *f* applied by placement *p* to crop *cro* in grid *g* in the year *y*. *Harea*_*cro,y,g*_ (ha) is harvested area of crop *cro* in grid *g* in the year *y*. *Nrate*_*cro,y,g*_ (kg N ha^−1^) is N application rate (N application amount per harvested area) of crop *cro* in grid *g* in the year *y*. *FerRatio*_*f,y,g*_ (%) is the percentage of fertilizer *f* inputs to total N application amount in grid *g* in the year *y*. *Placement*_*cro,f,p,y,g*_ (%) is the percentage of surface placement *p* (or deep placement) inputs to total inputs of fertilizer *f* applied to crop *cro* in grid *g* in the year *y*. It’s noteworthy that crops were aggregated into 21 crop groups according to Zhan *et al*.^29^. The detailed methods for generating each individual item were described as below, respectively. The workflow of methods is presented in Fig. 1. Main variables used in equations see Table 1. Main data used for developing this new crop-specific N fertilization dataset are listed in Table 2.

**Table 1:** Main variables used in methods.

Variables	Definition	Sources	Units
*cro*	Crop groups defined in this study	Zhan *et al*.^29^	—
*cro2*	Crop groups defined in FUBC	FUBC^33^	—
*f*	Fertilizer types	Developed by authors	—
*p*	Placements	Developed by authors	—
*y*	Year	Developed by authors	—
*g*	Grid	Developed by authors	—
*i*	County/district	Developed by authors	—
*j*	Country	Developed by authors	—
*m*	Country or region	Developed by authors	—
*Namount*_*cro,f,p,y,g*_	N application amount	Developed by authors	kg N
*Nrate*_*cro,y,g*_	Total N application rate	Developed by authors	kg N ha^−1^
*Harea*_*cro,y,g*_	Harvested area	Developed by authors	ha
*FerRatio*_*f,y,g*_	The percentage of fertilizer *f* inputs to total N application amount	Developed by authors	%
*Placement*_*cro,f,p,y,g*_	The percentage of surface placement (or deep placement) inputs to total application of fertilizer *f*	Developed by authors	%
*HareaM*_*cro,2000,i*_	Harvested area around 2000 from the EARTHSTAT	EARTHSTAT^28^.	ha
*HareaFAO*_*cro,y,j*_	Harvested area from the FAOSTAT	FAOSTAT^20^	ha
*Carea*_*upland,y,g*_	Cropland area for upland crops from the HYDE3.2	HYDE3.2^31^	ha
*Carea*_*paddy rice,y,g*_	Cropland area for paddy rice from the HYDE3.2	HYDE3.2^31^	ha
*NamountFAO*_*f,y,i*_	N application amount from the FAOSTAT	FAOSTAT^20^	kg N
*NamountW*_*f,y,i*_	N application amount from Wang *et al*.	Wang *et al*^24^.	kg N
*NrateZ*_*cro,f,2000,i*_	N application rate in the year 2000 from Zhan *et al*.	Zhan *et al*^29^.	kg N ha^−1^
*NconFUBC*_*cro2,SN,y0,j*_	SN fertilizer consumption from FUBC	FUBC^33^	kg N ha^−1^
*FerIFA*_*f,y,m*_	N application amount of fertilizer *f* from the IFASTAT	IFASTAT^21^	kg N
*FerIFA*_*total,y,m*_	N application amount of total synthetic fertilizers from the IFASTAT	IFASTAT^21^	kg N
*Placement*_*cro,f,deep,y,g*_	The percentage of deep placement N inputs to total N inputs	Developed by authors	%
*Placement*_*cro,f,surface,y,g*_	The percentage of surface placement N inputs to total N inputs	Developed by authors	%
*NoTill*_*cro,y,g*_	Non-tillage ratio	Developed by authors	%

Note: - means dimensionless.

**Table 2:** Main data used for developing this new N fertilization dataset.

Data name	Year	Spatial resolution	Reference
Harvested area for 175 crops	average of 1997–2003	5 min by 5 min	EARTHSTAT^28^
Harvested area for 175 crops	1961–2020	Country level	FAOSTAT^20^
Gridded cropland area	1960–2017	5 min by 5 min	HYDE3.2^31^
Nitrogen application rate for 21 crop groups	2000	5 min by 5 min	Zhan *et al*.^29^
Nitrogen application to cropland	1961–2014	5 min by 5 min	Wang *et al*.^24^
Nitrogen application amount	2014–2020	Country level	FAOSTAT^20^
Crop-specific nitrogen application amount	2006, 2007, 2010, 2014, 2018	Country or region	FUBC^33^
Fertilizer inputs for different fertilizer types	1961, 1973–2020	Country or region	IFASTAT^21^
National GDP	1961–2020	Country level	Word Bank^37^
Population	1961–2020	Country level	FAOSTAT^20^
Cropland area	1961–2020	Country level	FAOSTAT^20^
No-tillage area of China	1980–2017	Province level	CAMI^43^
No-tillage area by USA	1989–2008	State level	CTIC^44^
No-tillage area for 75 countries	1989–2018	Country level	FAOSTAT,EUROSTAT^20,27^
Harvested area of irrigated crops and rainfed crops	2005,2010	5 min by 5 min	SPAM2005,SPAM2010^45,46^
Field size	2015	1 km by 1 km	Lesiv *et al*.^47^
Income level	1987–2018	Country level	Word Bank^37^
Water erosion	2001,2013	5 min by 5 min	Borrelli *et al*.^48^
Potential evapotranspiration and precipitation	1961–2020	0.5° by 0.5°	CRU^30^

### Crop-specific harvested areas

The gridded crop-specific harvested area (ha) for the year 1961–2020 (*Harea*_*cro,y,g*_) was integrated from gridded harvested area in the year 2000 from the EARTHSTAT^28^ and country-level statistics of crop-specific harvested area during the period of 1961–2020 from the FAOSTAT^20^. The historical areas of arable land and permanent crops (here referred to cropland areas), were also included from the History Database of the Global Environment (HYDE version 3.2)^31^ to identify the spatial distributions of cropland and to map the statistics of harvested areas onto a grid.

The gridded harvested area from the EARTHSTAT^28^ is relatively detailed and covers a wide range of crop types, which was developed based on agricultural census and survey information of 175 crops from the 22,050 political units reasonably obtainable for 206 countries during 1997–2003. Thus, we developed crop-specific gridded harvested area based on this dataset. First, the harvested area of 136 crops (accounting for 92.9% of total harvested area) from the EARTHSTAT^28^ were integrated into 21 crop groups and administrative units (i.e., county/district, a lower level subdivision than state or province) according to Zhan *et al*.^29^ and political boundaries from the GADM^32^, respectively. Note that, arable forage crops (such as green maize, alfalfa) or temporary and permanent grasslands are not included in this study. Details for crop information see Table 3.

**Table 3:** Crop Designations.

Crop name in this study	Crop name in the EARTHSTAT	Crop name in the FUBC
Rice	rice	Rice
Maize	maize	Maize
Wheat	wheat	Wheat
Barley	barley	Other cereals
Sorghum	sorghum	
Millet	millet	
Rye	rye	
Sugarbeet	sugarbeet	Sugar crops
Sugarcane	sugarcane	
Oil palm	oilpalm	Oil palm
Sunflower	sunflower	Other oil crops
Rapeseed	rapeseed	
Groundnut	groundnut	
Potato	potato	Other crops
Sweetpotato	sweetpotato	
Cassava	cassava	
Cotton	cotton	Cotton
Soybean	soybean	Soybean
Vegetables	artichoke,asparagus,cabbage,carrot,cauliflower,chilleetc,cucumberetc,eggplant,garlic,greenbean,greenbroadbean,greencorn,greenonion,greenpea,lettuce,melonetc,mushroom,okra,onion,pumpkinetc,spinach,stringbean,tomato,vegetablenes,watermelon	Vegetables and fruits
Fruits	apple,apricot,avocado,banana,berrynes,blueberry,carob,cashewapple,cherry,citrusnes,cranberry,currant,date,fig,fruitnes,gooseberry,grape,grapefruitetc,kiwi,lemonlime,mango,orange,papaya,peachetc,pear,persimmon,pineapple,plantain,plum,quince,rasberry,sourcherry,stonefruitnes,strawberry,tangetc,tropicalnes	
Other crops	abaca,agave,almond,aniseetc,areca,bambara,bean,brazil,broadbean,buckwheat,canaryseed,cashew,castor,cerealnes,chestnut,chickpea,chicory,cinnamon,clove,cocoa,coconut,coffee,cowpea,fibrenes,flax,fonio,ginger,gums,hazelnut,hemp,hempseed,hop,jute,jutelikefiber,kapokfiber,kapokseed,karite,kolanut,lentil,linseed,lupin,mate,melonseed,mixedgrain,mustard,nutmeg,nutnes,oats,oilseednes,olive,pea,pepper,peppermint,pigeonpea,pimento,pistachio,poppy,pulsenes,pyrethrum,quinoa,ramie,rootnes,rubber,safflower,sesame,sisal,spicenes,sugarnes,taro,tea,tobacco,triticale,tung,vanilla,vetch,walnut,yam,yautia	Other crops

Then, the national harvested area from the FAOSTAT^20^ was then used to extend the harvested area from the EARTHSTAT^28^ (as a reference map for the distribution of harvested area) in the year 2000 to the period of 1961–2020. The harvested area can be extended by the following equation:

2 $$\begin{array}{c}Hare{a}_{cro,y,i}=Harea{M}_{cro,2000,i}\times \frac{HareaFA{O}_{cro,y,j}}{HareaFA{O}_{cro,2000,j}}\end{array}$$

where, *Harea*_*cro,y,i*_ is harvested area of crop *cro* in the county/district *i* in the year *y*. *HareaM*_*cro,2000,i*_ is harvested area in the county/district *i* around the year 2000 from the EARTHSTAT^28^. *HareaFAO*_*cro,y,j*_ is harvested area of crop *cro* in the country *j* from the FAOSTAT^20^. Here, we assumed that the annual changes within each county/district (*i*) of country (*j*) were consistent with the annual changes in that respective country. The harvested area of the countries, which experienced political disintegration, was distributed to individual newly-formed countries using the ratio derived from the harvested area in the first year after disintegration.

Thirdly, the crop-specific harvested area for each county/district during the period of 1961–2020 (*Harea*_*cro,y,i*_) was mapped onto a grid by spatially disaggregating it using the dynamic cropland distribution from the HYDE3.2^31^. The HYDE3.2^31^ provided distribution and area croplands for a long time series (annual time-step after the year 2000 to 2017 and a decadal time-step during 1700–2000). The mapping of the gridded crop-specific harvested area was in proportion to the cropland area of each grid in each county/district, which can be calculated as below:

3 $$\begin{array}{ccc}Hare{a}_{cro,y,g} & = & \left\{\begin{array}{lc}Hare{a}_{cro,y,i}\times \frac{Care{a}_{rice,y,g}}{Care{a}_{rice,y,i}} & if\;cro=rice\\ Hare{a}_{cro,y,i}\times \frac{Care{a}_{upland,y,g}}{Care{a}_{upland,y,i}} & if\;cro=rest\;of\;crops\end{array}\right.\end{array}$$

where, *Harea*_*cro,y,g*_ is the harvested area of crop *cro* in grid *g*. *Carea*_*rice,y,g*_ and *Carea*_*upland,y,g*_ are cropland area of rice and upland for grid *g* from the HYDE3.2^31^, respectively. *Carea*_*rice,y,i*_ and *Carea*_*upland,y,i*_ are total calculated cropland area of rice and upland for county/district *i* from the HYDE3.2^31^. Due to the cropland area from the HYDE3.2^31^ was available only up to the year 2017 and at decadal time-step before the 1990s, we assumed that the cropland area after the year 2017 was the same as that in 2017 and the cropland area before the year 2000 was constant in each decade. Lastly, to be comparable with that from the FAOSTAT^20^, the disaggregated gridded dataset was matched by the total crop-specific harvested area of each country from the FAOSTAT^20^.

### Crop-specific N application rate

The gridded crop-specific N application rate (including synthetic N fertilizers (SN), crop residues (CR) and manure (MA)) for the year 1961–2020 (*Nrate*_*cro,y,g*_) was derived from the gridded N application amount in 1961–2014 developed by Wang *et al*.^24^, the country level N consumption for agriculture from the FAOSTAT^20^ (including SN, CR, and MA), the country-level crop-specific fertilizer consumption from FUBC^33^ and the N application rate for 21 crop groups from Zhan *et al*.^29^.

Due to its high spatial and temporal resolution, the gridded N application amount in 1961–2014 provided by Wang *et al*.^24^ was used as a reference dataset to develop the crop-specific N application rate. Firstly, the gridded N application amount (divided into SN, CR, and MA) from Wang *et al*.^24^ was integrated into the GADM administrative units^32^ such as county, district or equivalent that is a lower level subdivision than state or province. Then, SN, MA, and CR application amount from the FAOSTAT^20^ spanning the period from 1961–2020 were utilized to extend and calibrate the above aggregated N application amount. Since the N application amount from the FAOSTAT^20^ includes both the cropland use and the other agricultural uses (e.g., pasture), we separated fertilizer use applied to croplands for each county/district based on the country-scale crop-wise proportion^34^, by assuming the same proportion of cropland use within a certain country. The calculation was described as below:

4 $$\begin{array}{c}Namount{W}_{f,y,i}=Namount{W}_{f,2014,i}\times \frac{NamountFA{O}_{f,y,j}}{NamountFA{O}_{f,2014j}}\;if\;2015\le y\le 2020\end{array}$$

Where *NamountW*_*f,y,i*_ is N application amount of fertilizer *f* (SN, MA or CR) for county/district *i* in the year *y* (from 2015 to 2020). *NamountW*_*f,2014,i*_ is N application amount of fertilizer *f* (SN, MA or CR) developed by Wang *et al*.^24^ for county/district *i* in 2014. *NamountFAO*_*f,y,j*_ and *NamountFAO*_*f,2014,j*_ is application amount of fertilizer *f* (SN, MA or CR) from FAOSTAT^20^ for country *j* in the year *y* and 2014.

Note that, total N application amount from Wang *et al*.^24^ was on average 12% lower than the FAOSTAT^20^ that removed the pasture synthetic-fertilizer application, due to the reduced amount of SN application. Thus, we corrected SN application amount from Wang *et al*.^24^ to the FAOSTAT^20^. CR and MA application amount were consistent with Wang *et al*.^24^. Then, the total N application amount (*Namount*_*f,y,i*_) of this dataset was obtained and the calculation was described as below:

5 $$\begin{array}{c}Namoun{t}_{f,y,i}=\left\{\begin{array}{c}Namount{W}_{f,y,i}\;if\;f=CR\;or\;MA\\ Namount{W}_{f,y,i}\times \frac{NamountFA{O}_{SN,y,j}}{Namount{W}_{f,y,j}}\;if\;f=SN\end{array}\right.\end{array}$$

Then, since gridded crop-specific N application rate of SN, MA, and CR from Zhan *et al*.^29^ were only available in 2000, we assumed that the N application rate changed at the same ratio for all crops across all the time during 1961–2020 according to Zhang *et al*.^35^. We used the same calculation as Zhang *et al*.^35^ to calculate the N application rate of fertilizer *f* (SN, MA, and CR) applied to crop *cro* for county/district *i* in the year *y* (*Nrate*_*cro,f,y,i*_).

6 $$\begin{array}{c}Nrat{e}_{cro,f,y,i}=Nrate{Z}_{cro,f,2000,i}\times \frac{Namoun{t}_{f,y,i}}{{\sum }_{cro}Nrate{Z}_{cro,f,2000,i}\times Hare{a}_{cro,y,i}}\end{array}$$

where *Nrate*_*cro,f,y,i*_ is N application rate of fertilizer *f* (SN, MA, CR) applied to crop *cro* for county/district *i* in the year *y*. *NrateZ*_*cro,f,2000,i*_ is N application rate of fertilizer *f* (SN, MA, CR) applied to crop *cro* for county/district *i* in the year 2000 from Zhan *et al*.^29^. *Namount*_*f,y,i*_ is N application amount of each county/district obtained from Eq. (5). *NamountW*_*f,y,i*_ and *Harea*_*cro,y,g*_ was obtained above.

Lastly, to minimize the uncertainties of crop-specific N application rate, we calibrated crop-specific N application rate after the year 2003 using country-level N synthetic fertilizer consumption reported by the Fertilizer Use By Crop (FUBC)^33^ for the years 2006, 2007, 2010, 2014 and 2018. Thus, the above-derived crop-specific synthetic N application rate (*Nrate*_*cro,SN,y0,i*_) multiplied with the crop-specific harvested area (*Harea*_*cro,y0,i*_) was used to achieve crop-specific N application amount, and then synthetic N crop-specific amount of each crop group belongs to crop group *cro2* was summed up by county/district *i* and corrected with the country-level N synthetic fertilizer consumption by crop group *cro2* from the FUBC^33^. The calculation was described below:

7 $$\begin{array}{c}Nrat{e}_{cro,f,y,i}=Nrat{e}_{cro,f,y,i}\times \frac{NconFUB{C}_{cro2,SN,y0,j}}{\sum _{cro,i}Nrat{e}_{cro,SN,y0,i}\times Hare{a}_{cro,y0i}}\\ \left\{\begin{array}{c}if\,\,2003\le y\le 2006,y0=2006\\ if\,\,2007\le y\le 2008,y0=2007\\ if\,\,2009\le y\le 2012,y0=2010\\ if\,\,2013\le y\le 2016,y0=2014\\ if\,\,y\ge 2017,y0=2018\end{array}\right.\end{array}$$

where *NconFUBC*_*cro2,SN,y0,j*_ is SN fertilizer consumption from FUBC^33^ to the 11 crop groups in the year 2006, 2007, 2010, 2014, 2018.

Finally, the county-level crop-specific N application rate (divided into SN, CR, and MA) summed up to generate the total N application rate and then was resampled into 5 arc-min resolution assuming that N application rate of each grid in a county/district was equal to the N application rate of this county/district, thus the crop-specific N application rate in each grid (*Nrate*_*cro,y,g*_) was obtained.

### N fertilizer types

The gridded percentage of different N fertilizer type inputs to total N application amount for the year 1961–2020 (*FerRatio*_*f,y,g*_) was integrated from country- and continent-scale annual fertilizer consumption surveys provided by the IFASTAT^21^ and N application amount (*Namount*_*f,y,i*_) developed above. We sorted N fertilizers into 13 types, include anhydrous ammonia (AA), ammonium nitrate (AN), ammonium sulphate (AS), calcium ammonium nitrate (CAN), nitrogen solutions (NS), other N straight (ONS), Urea, ammonium phosphate (AP), N K compounds (NK), N P K compounds (NPK), other NP (ONP), crop residues (CR) and manure (MA).

Consumption of each synthetic fertilizer type was available from both the IFASTAT^21^ and the FAOSTAT^20^. However, about 5.87% and 59.3% of the values from the IFASTAT^21^ and the FAOSTAT^20^, respectively, was missing. Hence, we chose to apply fertilizer consumption data from the IFASTAT due to the lesser missing values. Consumption of SN fertilizers for 1962–1972 from the IFASTAT is missing for all countries and regions. Moreover, the data is also missing from countries that have experienced political disintegration, especially in the years following it. As for data from the FAOSTAT^20^, missing data spread all over the period 1961–2020, especially for the developing countries. The missing data from the IFASTAT^21^ was filled by the multiple data imputation method (e.g., “Amelia”) for time-series cross-section datasets proposed by King *et al*.^36^, which were used by Nishina *et al*.^23^ to fill the missing fertilizer consumption data from the FAOSTAT. Covariates (e.g., the national GDP (from the World Bank^37^), population (from the FAOSTAT^20^), and cropland area (from the FAOSTAT^20^)) were chosen to apply the “Amelia” algorithm for imputation of the missing values as Nishina *et al*.^23^. R package “Amelia” was used for statistical imputation and settings, which is ideal in handling national-based time-series and cross-sectional data with advanced statistical imputation protocol^23^. More details see Nishina *et al*.^23^.

Then, the calculation for the percentage of each N fertilizer type inputs to total N application amount can be sorted into two classifications. For crop residues and manure, the percentage can be directly obtained with the ratio of crop residue and manure inputs amount and total N application amount. For the remaining 11 synthetic fertilizers, the percentage was calculated through multiplying the consumption proportion of each synthetic fertilizer to the total synthetic N fertilizer from the IFASTAT by the proportion of synthetic fertilizer inputs to total N application amount obtained above.

The percentage of each fertilizer consumption can be calculated as following equations:

8 $$\begin{array}{c}FerRati{o}_{f,y,i}=\left\{\begin{array}{cc}\frac{Ninput{s}_{f,y,i}}{Ninput{s}_{total,y,i}} & f=CR,MA\\ \frac{FerIF{A}_{f,y,m}}{FerIF{A}_{total,y,m}}\times \frac{Ninput{s}_{SN,y,i}}{Ninput{s}_{total,y,i}} & f\;belongs\;to\;SN\end{array}\right.\end{array}$$

where, *FerRatio*_*f,y,i*_ is the percentage of fertilizer *f* inputs to total N application amount in the county/district *i* in the year *y*. *Namount*_*f,y,i*_, *Namount*_*SN,y,i*_*, Namount*_*total,y,i*_ was generated above, which represents N application amount of fertilizer *f* (MA or CR), SN and all fertilizers for county/district *i* in the year *y*, respectively. *FerIFA*_*f,y,co*_ and *FerIFA*_*total,y,co*_ is the consumption amount of synthetic fertilizer *f* and *total* synthetic fertilizer in the country or region *m* in the year *y* from the IFASTAT^21^, respectively.

Lastly, the percentages of each fertilizer were resampled into 5 arc-min resolution to obtain the percentages of each fertilizer in each grid (*FerRatio*_*f,y,g*_).

### N application placement

The gridded percentage of different N fertilizer types applied with deep placement (or surface placement) inputs to total input applied to different crop (*Placement*_*cro,f,deep,y,g*_ and *Placement*_*cro,f,surface,y,g*_) for the year 1961–2020 was quantified based on fertilizer type, tillage practice, and base-topdressing application ratio, as referred to Zhan *et al*.^29^. and can be calculated by the following equations.

9 $$\begin{array}{c}Placemen{t}_{cro,f,deep,y,g}=\left\{\begin{array}{cl}1 & f={\rm{AA,\; AN}}\\ 0.7\times NoTil{l}_{cro,y,g} & f=Other\;fetilizer\\ 1-NoTil{l}_{cro,y,g} & f=CR,MA\end{array}\right.\end{array}$$

10 $$\begin{array}{c}Placemen{t}_{cro,f,surface,y,g}=1-Placemen{t}_{cro,f,deep,y,g}\end{array}$$

where, *Placement*_*cro,f,deep,y,g*_ and *Placement*_*cro,f,surface,y,g*_ is the percentage of fertilizer *f* applied with deep placement and surface placement inputs to total inputs applied to crop *cro* in grid *g* in the year *y*, respectively. *NoTill*_*cro,y,g*_ is non-tillage ratio for crop *cro* in grid *g* in the year *y*. 0.7 is base-topdressing application ratio from Nishina *et al*.^23^.

Anhydrous ammonia and N solutions, commonly injected, were placed deep or totally infiltrated into soils^38^. All manure and crop residues were assumed to be applied before sowing or transplanting, thus the incorporation of manure and crop residues was linearly correlated to the tillage fraction^39^. For the rest synthetic fertilizer, if it was applied as base fertilizers, the proportion of incorporation or deep application should be positively related to the fraction of non-tillage (zero tillage or direct drilling during planting) in high-income countries. Because in developed countries, the non-tillage was mainly achieved through mechanized seeding and fertilization practices^40,41^. However, when the synthetic fertilizer was applied during the top-dressing process if applicable, we assumed that broadcasting techniques were applied^42^. It is noteworthy that the gridded non-tillage fraction for each crop during the period of 1961–2020 (*NoTill*_*cro,y,g*_) was specially developed, including the obtain of national or sub-national non-tillage area and its downscaling to grid and crop.

We firstly calculated the national non-tillage fraction during the period of 1961–2020 with the regression model provided by Zhan *et al*.^29^, which fitted the relationship between the cropland area per capita of the rural population (i.e., a proxy of the spatial variation of labor availability for tillage activity) and country-level non-tillage fractions (46 countries from the FAOSTAT^20^ and EUROSTAT^27^ databases, as well as China (2000−2016)^43^ and the United States of America (1989−2008)^44^). National cropland area per capita of rural population for prediction can be obtained from the FAOSTAT^20^. Then the national non-tillage area can be calculated through multiplying the above-mentioned fraction and the cropland area from the HYDE3.2^31^. Specially, data of non-tillage area by province (or state) for China (2000–2016)^43^ and the USA (1989–2008)^44^ were publicly available and can be used directly; for the rest year, the sub-national non-tillage area was obtained by downscaling the above predicted national non-tillage area.

Then, the national non-tillage area was downscaled to grid and crop following the methods by Porwollik *et al*.^41^, with the gridded and country-scale datasets (including the harvested area of irrigated crops and rain-fed crops from the SPAM2005 and the SPAM2010^45,46^ field size^47^, income (World Bank^37^), water erosion^48^, and aridity (calculated by potential evapotranspiration and precipitation from the CRU TS V4.06^30^)). Firstly, potential non-tillage area was allocated based on crop type, field size and local income. The potential non-tillage area was assumed that derived from the cropland of 11 rain-fed annual crops (Soybean, Wheat, Maize, Barley, Rapeseed, Sunflower, Sorghum, Cotton, Milled, Groundnut, Vegetables) in grid cells reporting dominant large field size in low-income countries and all field sizes in high-income countries^49–51^.

Secondly, a logit model combined four predictors (including crop mix, field size, water erosion, and aridity) was developed to determine the probability of non-tillage occurrence per grid cell as well as the spatial distribution of country-level non-tillage area. Crop mix was defined as the ratio of the area sum of 11 non-tillage suitable crop types over the sum of total cropland area per grid cell, thus we assumed an increasing probability for non-tillage area occurrence in grid cells with an increasing cultivated area share of non-tillage suitable crop types. Farm size is highly positively correlated with the share of non-tillage area on arable land, with the coefficient of determination r^2^ = 0.66 (*p* < 0.001, slope = 0.116)^41^, thus non-tillage was thus assumed highly probable for cropland in grid cells with large fields (here serving as a spatial proxy for large farm size and mechanization). Besides, non-tillage was considered to be suitable for arable production under arid conditions, due to less aeration, more stable pores, and soil aggregates compared to soils managed with conventional tillage^50,51^. Moreover, erosion levels as allocation criteria, non-tillage was regarded as suitable for crop production in areas with elevated erosion levels to protect the soil surface^50,52–54^. The probability of no-tillage in a grid cell (*NTA*_*g*_) was derived through the following equation:

11 $$\begin{array}{c}NT{A}_{g}=\frac{1}{1+\exp (-{\sum }_{n=1}^{4}{k}_{n}(v{x}_{n}-xmi{d}_{n})}\end{array}$$

where *n* represents the input datasets of water erosion, aridity, crop mix (the ratio of sum of harvested area of no-tillage suitable crop types over the sum of total harvested area per grid cell), and field size (proxy for farm size), *k*_*n*_ refers to the slope value, *xmid*_*n*_ to the central points of each of the logit curves, and *vx*_*n*_ to grid cell values of the referring input dataset. Values of *k*_*n*_ *and xmid*_*n*_ are directly derived from Porwollik *et al*.^41^.

Thirdly, the abovementioned projected national or sub-national non-tillage area was downscaled to all grid cells suitable for non-tillage in this country based on the gridded probability. According to the non-tillage probability, the grid cell in this country was sorted in decreasing order, which was further selected as non-tillage until the added potential non-tillage area (calculated as grid area multiplied by the probability) reached the projected country-level non-tillage area threshold. Lastly, crop-specific non-tillage area per grid (*NoTill*_*cro,y,g*_) was attained by disaggregating the non-tillage area of per grid cell according to the proportion of harvested area occupied by each crop in this grid.

### Uncertainty analysis

A Monte Carlo simulation method was utilized to estimate the uncertainty for N application rate. To generate a proper estimate interval, here we mainly accounted for the uncertainties related to the assumption that the crop-specific N application rate changed at the same ratio for all crop types across all the time during 1961–2020. Thus, to estimate the uncertainty around the mean changing ratio of N application rate by crop and by region, we derived standard deviation, coefficient variations (CV) of each crop group for 24 regions or countries from 5-year (i.e. 2006, 2007, 2010, 2014, 2018) crop-specific fertilizer use reported by the Fertilizer Use By Crop (FUBC)^33^. Then, we run 1,000 iterations for each crop by randomly varying the annual change ratio by coefficient variations with normal distributions, so that the 95% prediction interval as well as standard deviation could be constructed. Undoubtedly, if there is a large inter-annual variation in N application rate for a particular crop in a region, the uncertainty associated with the estimated N application rate will be correspondingly high.

## Data Records

The gridded N application data by crops, fertilizer, and placement from 1961 to 2020 and the gridded harvested area (1961–2020) are available at National Tibetan Plateau/Third Pole Environment Data Center^55^. The dataset extend from 180°E to 180°W longitude and 90°S to 90°N latitude at a resolution of 5 arc-min for the temporal period 1961–2020 in standard WGS84 coordinate system. The dataset is provided in Hierarchical Data Format (HDF5) format, which can be read by many tools (i.e., IDL, MATLAB).

The gridded N application data by crops, fertilizer, and placement are stored in the file “N_rate_{‘Crop name’}_1961–2020.h5”, while {‘Crop name’} represents each crop group listed in Table 4. The file contains 26 records named by {‘Fertilizer type name’_‘Placement name’}, while {‘Fertilizer type name’_‘Placement name’} represents each fertilizer type with two kinds of fertilization placements listed in Table 4. In addition, to calculate the N application amount by crop, the gridded harvested area was summarized in the file “Harvested_area_1961–2020.h5”, containing 21 crop groups named by {‘Crop name’}. The unit of N application rate was kg N per hectare of harvested area and unit of the harvested area was hectare per 5-arc-min grid cell.

**Table 4:** List of data record names in the HDF5 files.

Crop name	Fertilizer type name	Placement name
Barley, Cassava, Cotton, Fruits, Groundnut, Maize, Millet, Oilpalm, Potato, Rapeseed, Rye, Rice, Sorghum, Soybean, Sugarbeet, Sugarcane, Sunflower, Sweetpotato, Vegetables, Wheat, Other crops	AA, AN, AS, CAN, NS, ONS, Urea, AP, NK, NPK, ONP, CR, MA	Surface, Deep

Note: AA, AN, AS, CAN, NS, ONS, AP, NK, NPK, ONP, CR, MA refers to anhydrous ammonia, ammonium nitrate, ammonium sulphate, calcium ammonium nitrate, nitrogen solutions, other N straight, ammonium phosphate, N K compounds, N P K compounds, other NP, crop residues and manure, respectively.

### Crop-specific N application

**Fig. 2:**
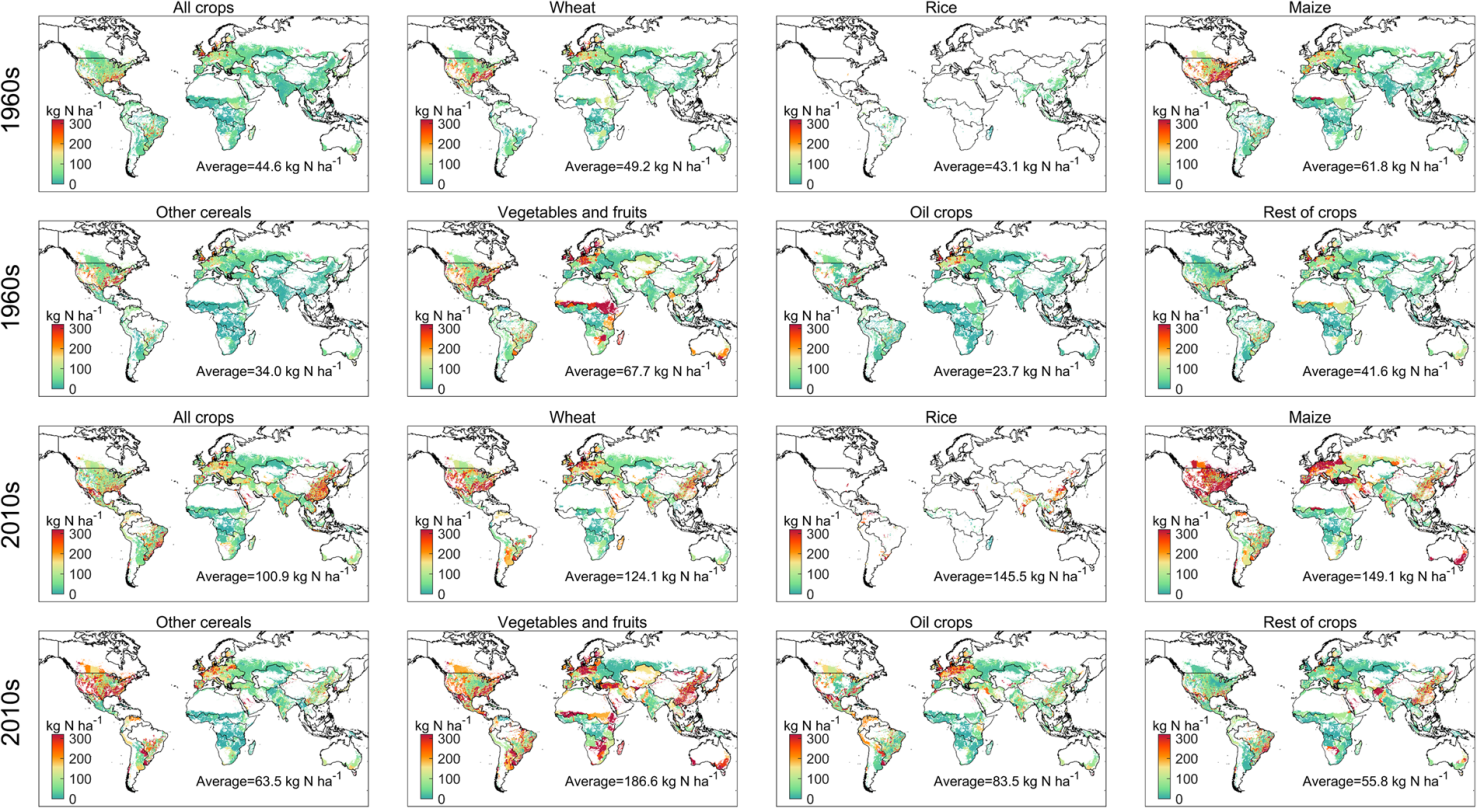
Spatial pattern of crop-specific N application rate (kg N per hectare of harvested area) applied to cropland in the 1960s and 2010s.

Crop-specific N application in the 1960s (average from 1961 to 1970) and 2010s (average from 2011 to 2020) were shown in Fig. 2. The average N application rate applied to cropland was 44.6 kg N ha^−1^ yr^−1^ in the 1960s, mainly in Europe and USA. Average N application rate for maize (49.2 kg N ha^−1^ yr^−1^), wheat (61.8 kg N ha^−1^ yr^−1^), vegetables and fruits (67.7 kg N ha^−1^ yr^−1^) were relatively higher than for other crops. In the 2010s, average N application rate increased to 100.9 kg N ha^−1^ yr^−1^, and the hotspots also shifted from Europe and the USA to China. The N application rate between 1961 and 2020 for rice, vegetables and fruits showed the highest growth rate, increasing by 237.6%, 175.6%, and 252.3%, respectively, compared to wheat (152.2%), maize (141.3%), other cereals (86.8%), and rest of crops (34.1%).

### N fertilizer types

**Fig. 3:**
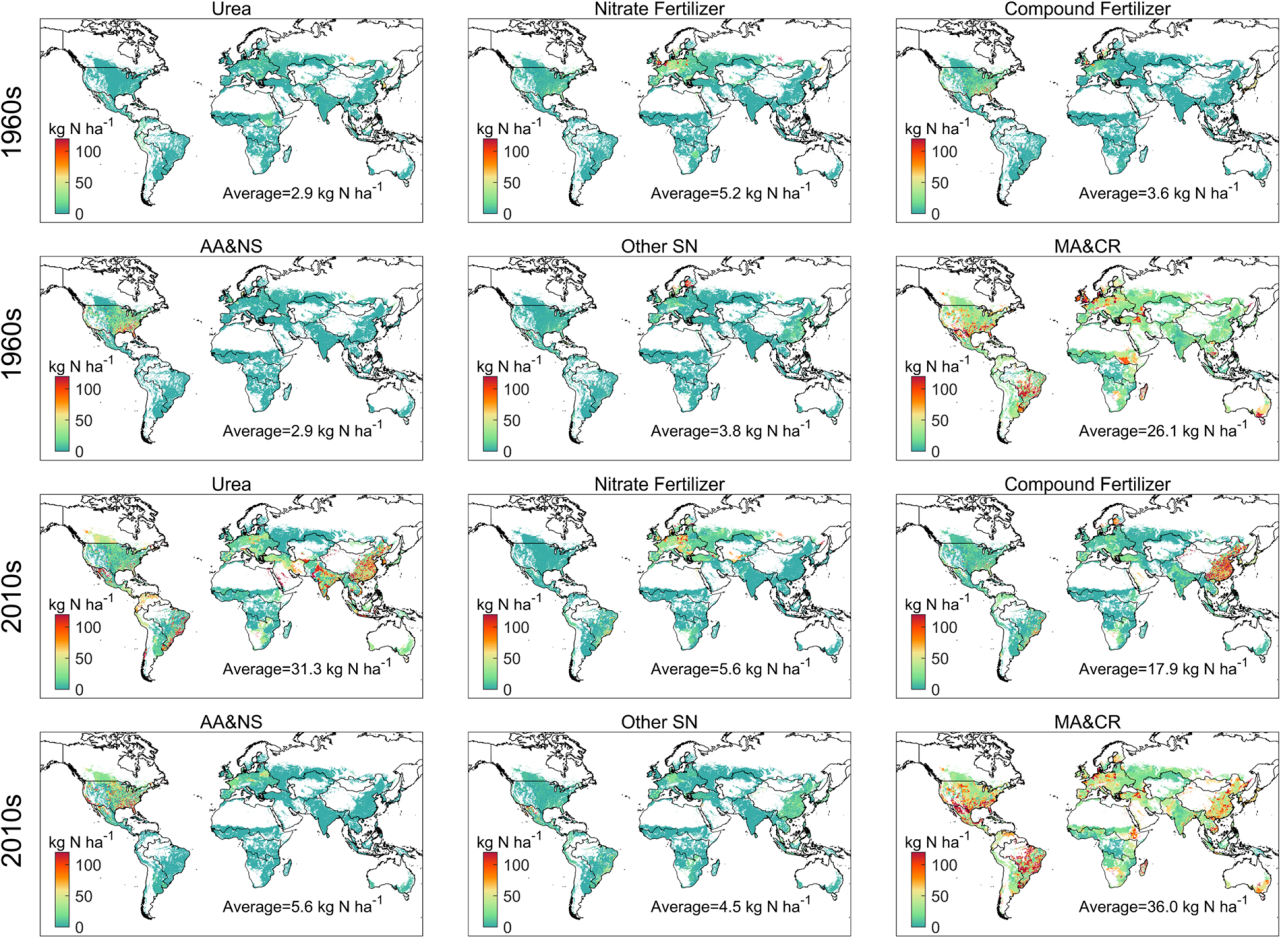
Spatial pattern of N application rate for different fertilizers (kg N per hectare of harvested area) applied to cropland. Fertilizers include Urea, nitrate fertilizers (ammonium nitrate (AN) and calcium ammonium nitrate (CAN)), compound fertilizers (ammonium phosphate (AP), N K compounds (NK), N P K compounds (NPK), other NP (ONP)), AA&NS (anhydrous ammonia (AA) and N solutions (NS)), other synthetic fertilizers (other N straight (ONS) and ammonium sulphate (AS)) and MA&CR (manure (MA) and crop residues (CR)).

The spatial pattern of the N application for each fertilizer was illustrated in Fig. 3. In the1960s, the N application rate of manure and crop residues was about 26.1 kg N ha^−1^ yr^−1^, and the hotspots were mainly in USA, Europe and south America. Hotspots of synthetic fertilizers varied depending on the fertilizer types. For urea, nitrate fertilizers and compound fertilizers, the hotspots were mainly distributed in Eastern Europe, while for other synthetic fertilizers were mainly in Southeastern China and Middle Europe. Anhydrous ammonia and N solutions were mainly applied in Middle USA. Both the N application rate and spatial pattern of different N-consumed fertilizers changed significantly after five decades, e.g., the contributions of synthetic fertilizers grew from 41.5% to 64.4%. Urea as the most important synthetic-N fertilizers, with an average N application rate more than 100 kg N ha^−1^ yr^−1^, shared 31.0% of global N fertilizer consumption in 2010s and were mainly distributed in China, India and Pakistan. The hotspots of compound fertilizers and manure and crop residues also expanded to China, while the hotspots of nitrate fertilizers, other synthetic fertilizers, anhydrous ammonia and N solutions didn’t change much.

### N application placement

**Fig. 4:**
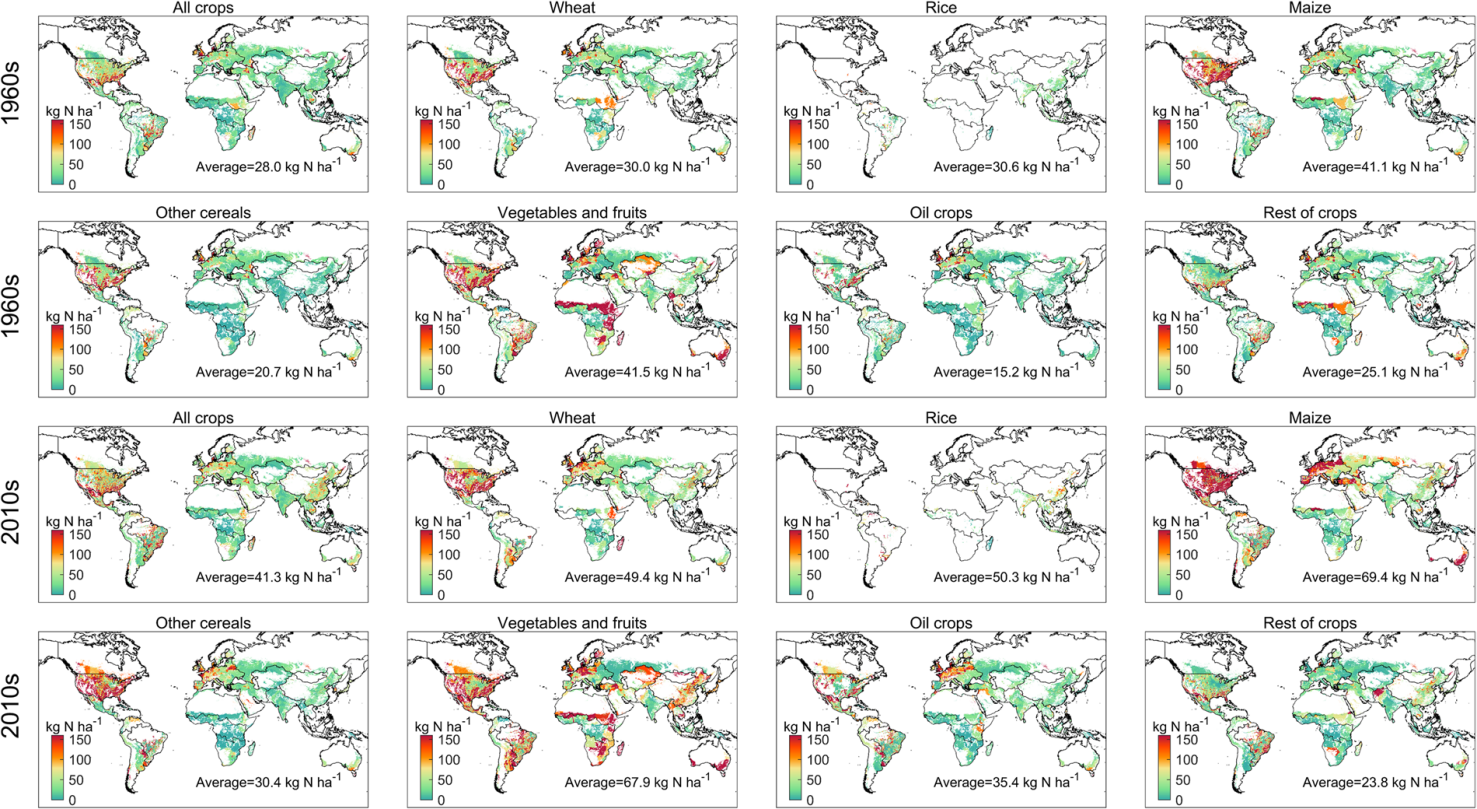
Spatial pattern of crop-specific N application rate (kg N per hectare of harvested area) applied by deep placement in the 1960s and 2010s.

Crop-specific N application rate by deep placement in the 1960s and 2010s were displayed in Fig. 4. The average N application rate applied by deep placement enhanced from 28.0 kg N ha^−1^ yr^−1^ in the 1960s to 41.3 kg N ha^−1^ yr^−1^ in the 2010s, while the hotspots were mainly in southern USA, southeast of South America, northwestern Europe and Australia during 1961–2020. N application rate by deep placement for most of crops has increased (64.7% for wheat, 64.4% for rice, 68.9% for maize, 46.9% for other cereals, 63.6% for vegetables and fruits and 132.9% for oil crops) during 1961–2020.

## Technical Validation

To check the spatiotemporal consistency and plausibility between the newly developed global crop-specific N fertilization dataset and previous datasets, we did a comprehensive comparison analysis, including the uncertainty analysis, direct validation analysis against various available data, etc.

**Fig. 5:**
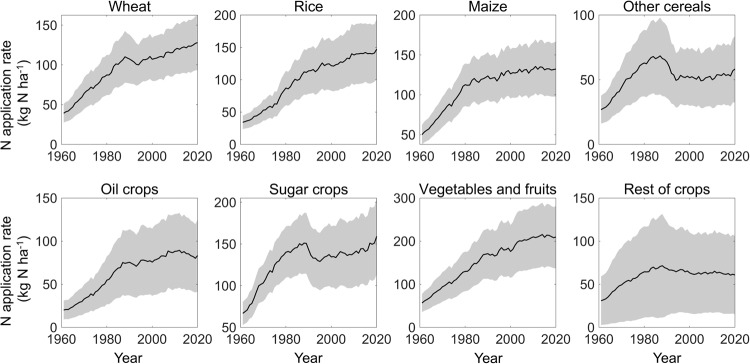
Uncertainties of global N application rate (kg N ha^−1^) for 8 crop groups. The grey color area in each panel shows the 95% confidence interval of N application rate estimates by Monte Carlo simulations, whereas the average value is presented by a black line.

**Fig. 6:**
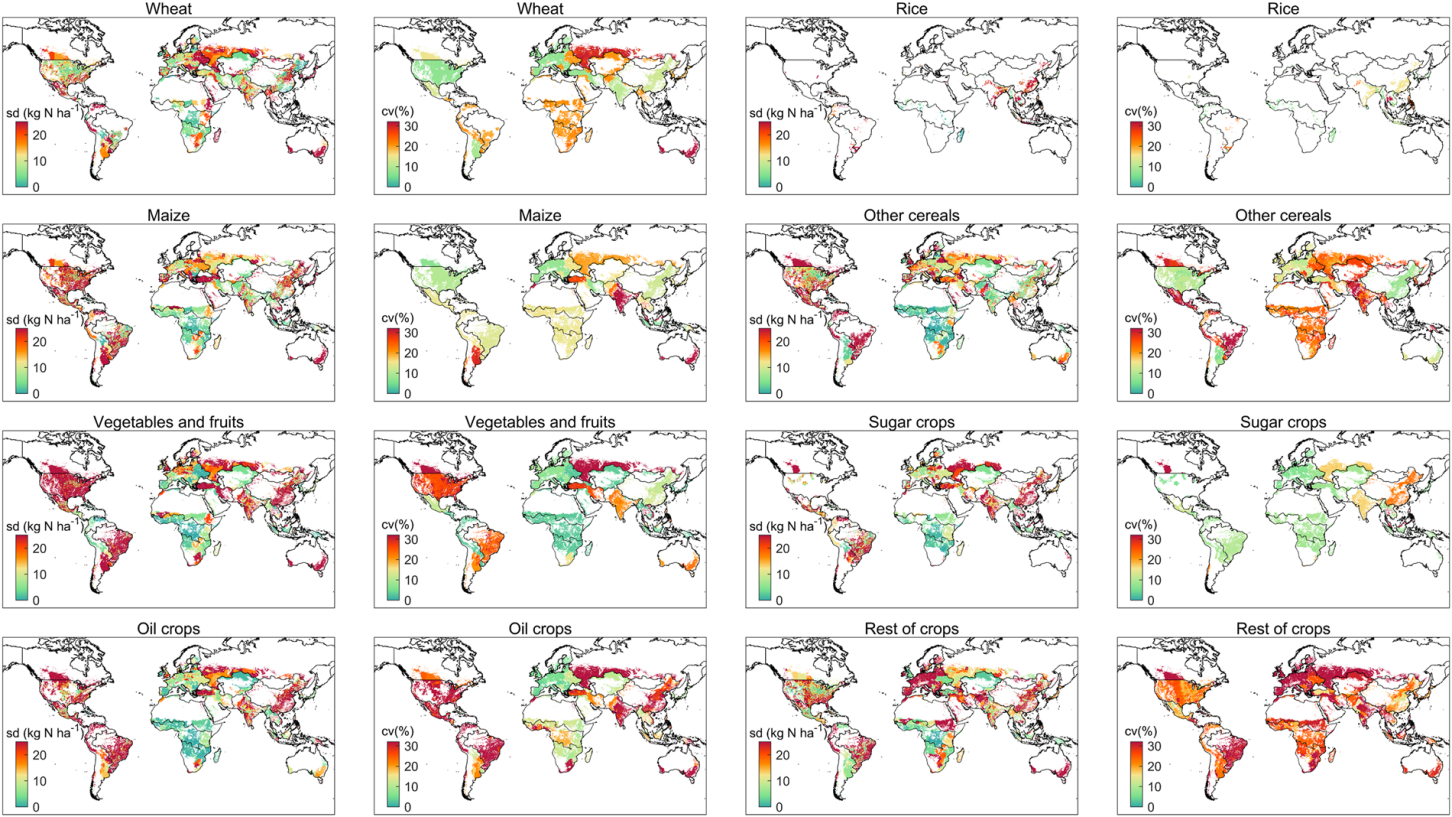
Spatial pattern of uncertainties related to N application rate (kg N ha^−1^) for 8 crop groups in 2010s.

Firstly, we utilized Monte Carlo simulations to quantify the uncertainties of crop-specific N application rate derived from the assumption, that the N application rate changed at the same ratio for all crops all the time. The 95% confidence intervals of crop-specific N application rate by crop, regions, and years can be gained, and are shown in Figs. 5, 6. Globally, uncertainties of wheat, rice, maize, sugar crops and vegetables&fruits at 95% confidence interval accounted for ±27.9%, ±31.5%, ±24.4%, ±25.1%, ±35.0% of average N application rate during 1961–2020, respectively (Fig. 5). Uncertainties of other cereals, oil crops, and rest of crops were larger (±42.0%, ±50.8% and ±80.0%), however, the three together only accounting for 34.9% of the total N application amount (Fig. 5). In addition, the spatial patterns of uncertainties related to the crop-specific N application rate were also generated (Fig. 6). Spatial heterogeneity of uncertainties was striking, regardless of crop types. For the vast majority of regions, the standard deviation (SD) of crop-specific N application rate were within acceptable limits, but in specific regions and for specific crops, the SD of the N application rate exceeded 20 kg N ha^−1^ yr^−1^ (Fig. 6). However, it is worth noting that N application rate tended to be higher in these regions and crops, such as China, Russia, and Australia for wheat; China and South Asia for rice; and the USA, China, and South America for maize. As a result, the coefficient of variation (CV) for these regions is relatively low, usually below 20%, despite the high standard deviation. Nevertheless, regions with both high SD and high CV, such as vegetables and fruits in USA, Russia and South America; maize in India and Southwest America; sugar crops in China, may have relatively large uncertainties, and caution should be exercised when using the N application rate of these crop groups in these regions. Despite the large uncertainties in certain regions and crop groups, it is important to note that the regions with large uncertainties (>20 kg N ha^−1^) and large CV (>25%) only contributed 8.9% of the total N application amount due to limited crop cultivation in these regions. This means that although we made very ambitious assumptions, the result looks reasonable due to the relatively low overall uncertainty.

Crop-specific N application rate from 2003 onwards was adjusted to match the crop-specific N fertilizer use from the FUBC^33^ report, which likely reduced uncertainties after 2000. As for the year before 2000, country-level fertilizer use data from the first to fifth FUBC report for the years 1978–2001 were extracted to validate the data. To determine the differences in the N application rate from this dataset with respect to the FUBC^33^ report, we calculated the country-wise relative difference *Dif* (%) as equation below:

12 $$\begin{array}{c}Di{f}_{cro,y,j}=\frac{Nrat{e}_{cro,y,j}-Nrate\_FUB{C}_{cro,y,j}}{Nrat{e}_{cro,y,j}}\times 100\end{array}$$

Where *Nrate*_*cro,y,j*_ is N application rate of crop *cro* in the country *j* in the year *y*. *Nrate_FUBC*_*cro,y,j*_ is corresponding N application rate from FUBC^33^.

**Fig. 7:**
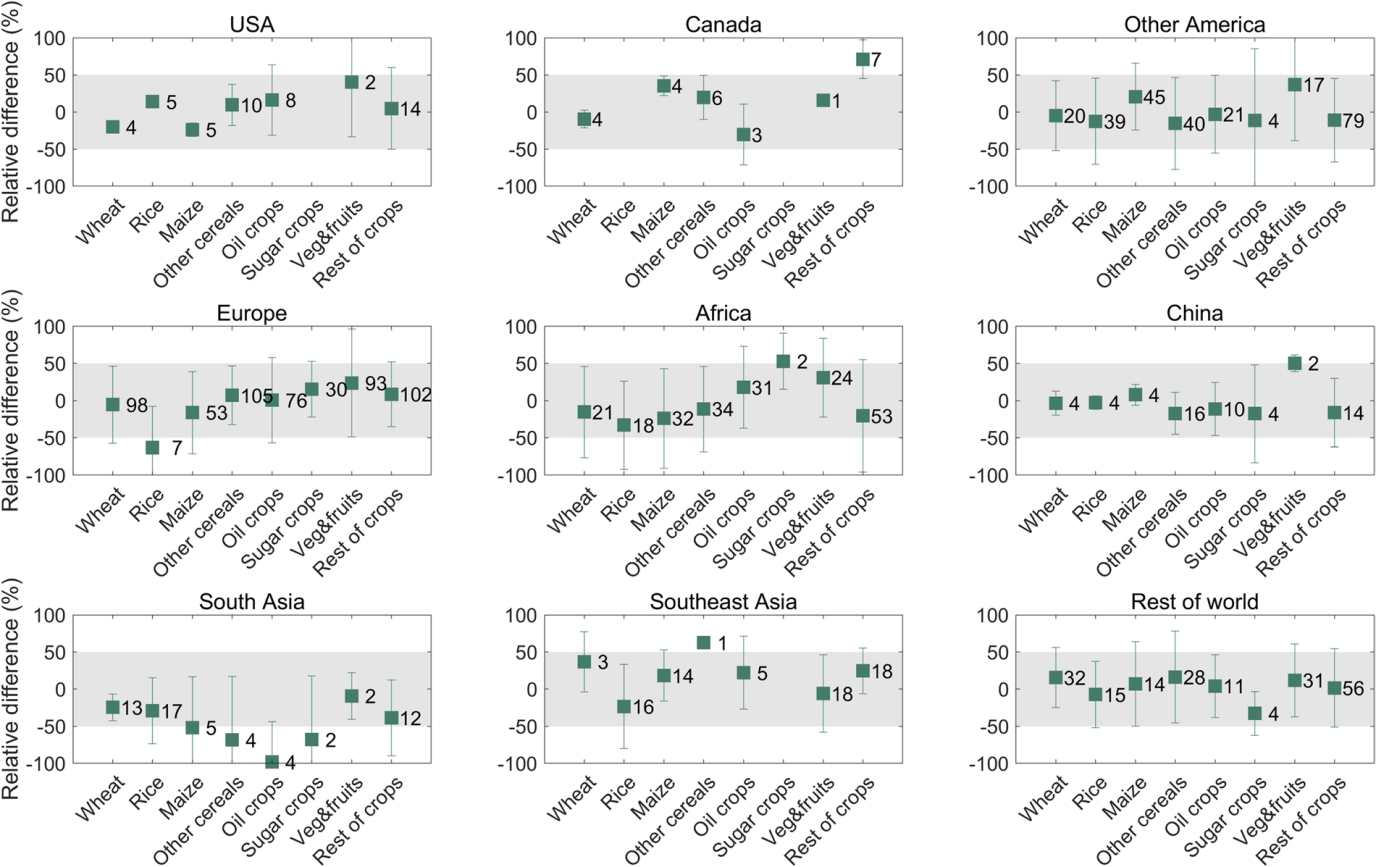
Relative differences between crop-specific N application rate in this dataset with data from the first to fifth FUBC report for the year during 1978–2001. The box plots show the range of relative differences, whereas numbers besides the box plots represents sample size of country-year.

The mean and SD for *Dif* of 8 crops groups and 9 countries or regions were presented here (Fig. 7). In most of the regions except for South Asia, the mean *Dif* between our estimates and the FUBC report was contained in the range of ±50%. Large values of the mean *Dif* were observed for some crops, such as vegetables and fruits in China (*Dif* = 50.4%), other crops in Canada (*Dif* = 70.9%), sugar crops in Africa (*Dif* = 53.0%), rice in Europe (*Dif* = −63.0%). N application rate for South Asia in this study might be underestimated due to most of the mean *Dif* for 8 crop groups lower than 0.

**Fig. 8:**
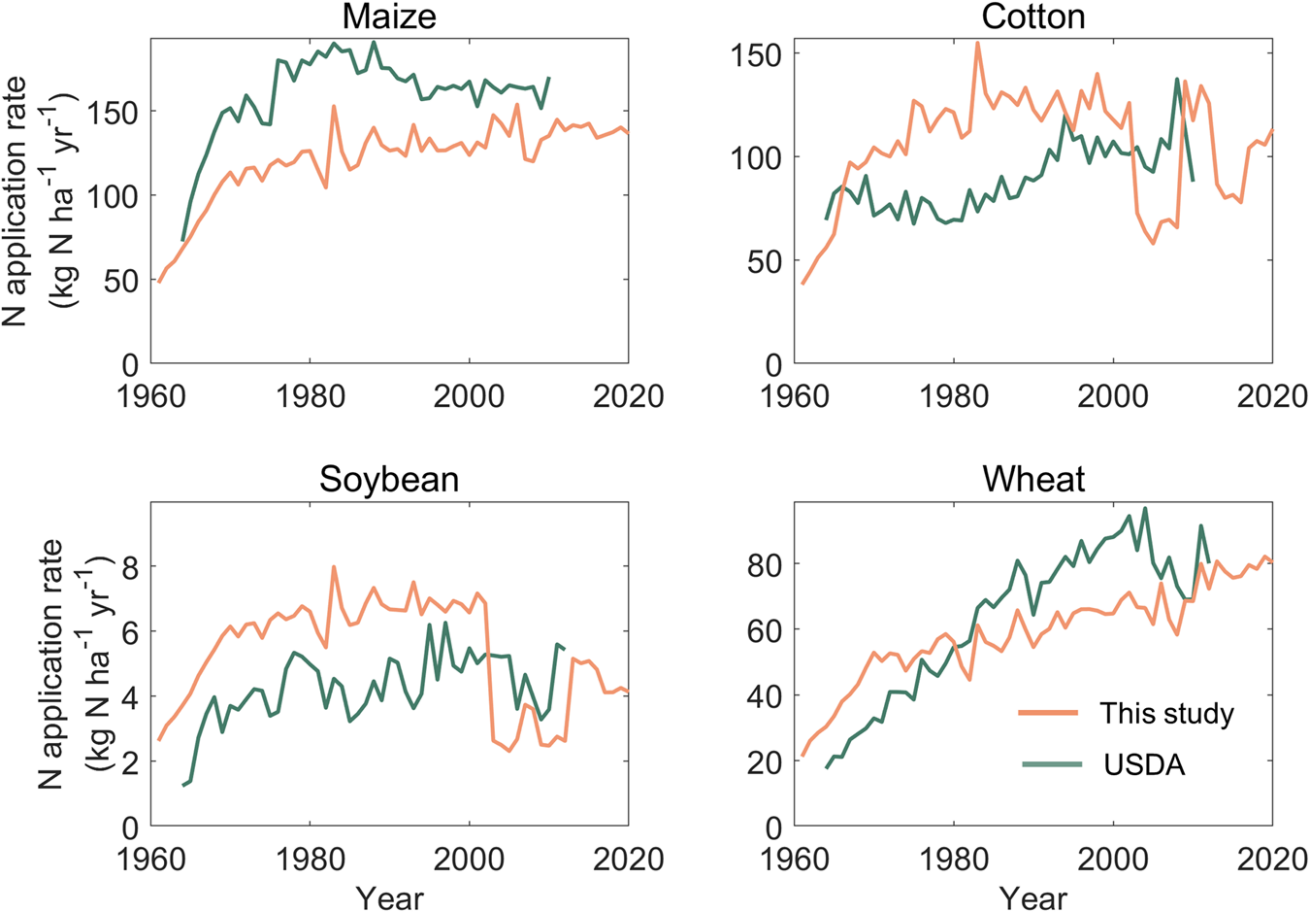
A comparison of N application rate between data reported by the United States Department of Agriculture (USDA) and this dataset.

**Fig. 9:**
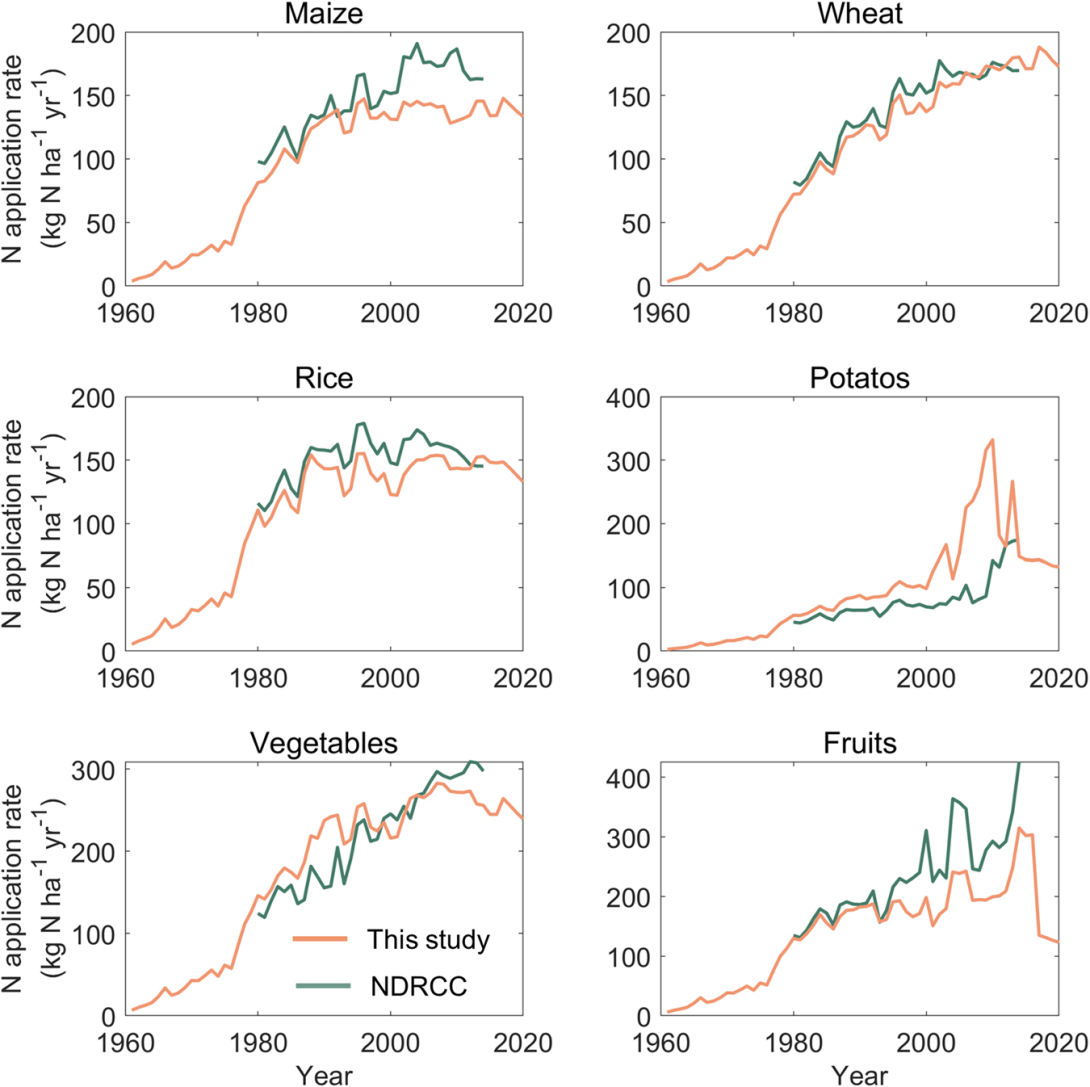
A comparison of N application rate between data reported by Cost and Income of Chinese Farm Produce (NDRCC) and this dataset.

Finally, we evaluated the reasonability of our results on a regional scale, with the historical N application statistics of USA and China from the United States Department of Agriculture (USDA)^56^ and the Cost and Income of Chinese Farm Produce (NDRCC 2003, 2022^57,58^), respectively. The N application rate for wheat in this study was relatively close to the USDA record (Fig. 8). However, for the USA, the N application rate for maize was 32.8% lower than the USDA record during 1964–2018, whereas those for cotton and soybean were 24.1% and 35.3% higher during 1964–2000, respectively. For China, our estimates for wheat, rice, maize, and potatoes are generally in agreement with the NDRCC record, with differences within the range of ±20% (Fig. 9). The estimated N application rate for vegetables and fruits were close to the records from the NDRCC before the year 2000, but after that, it was significantly lower (−13.8% and −36.4%). In general, the narrow differences between this study and regional statistic reflects that our crop-specific N application rate is relatively reasonable in regions.

**Fig. 10:**
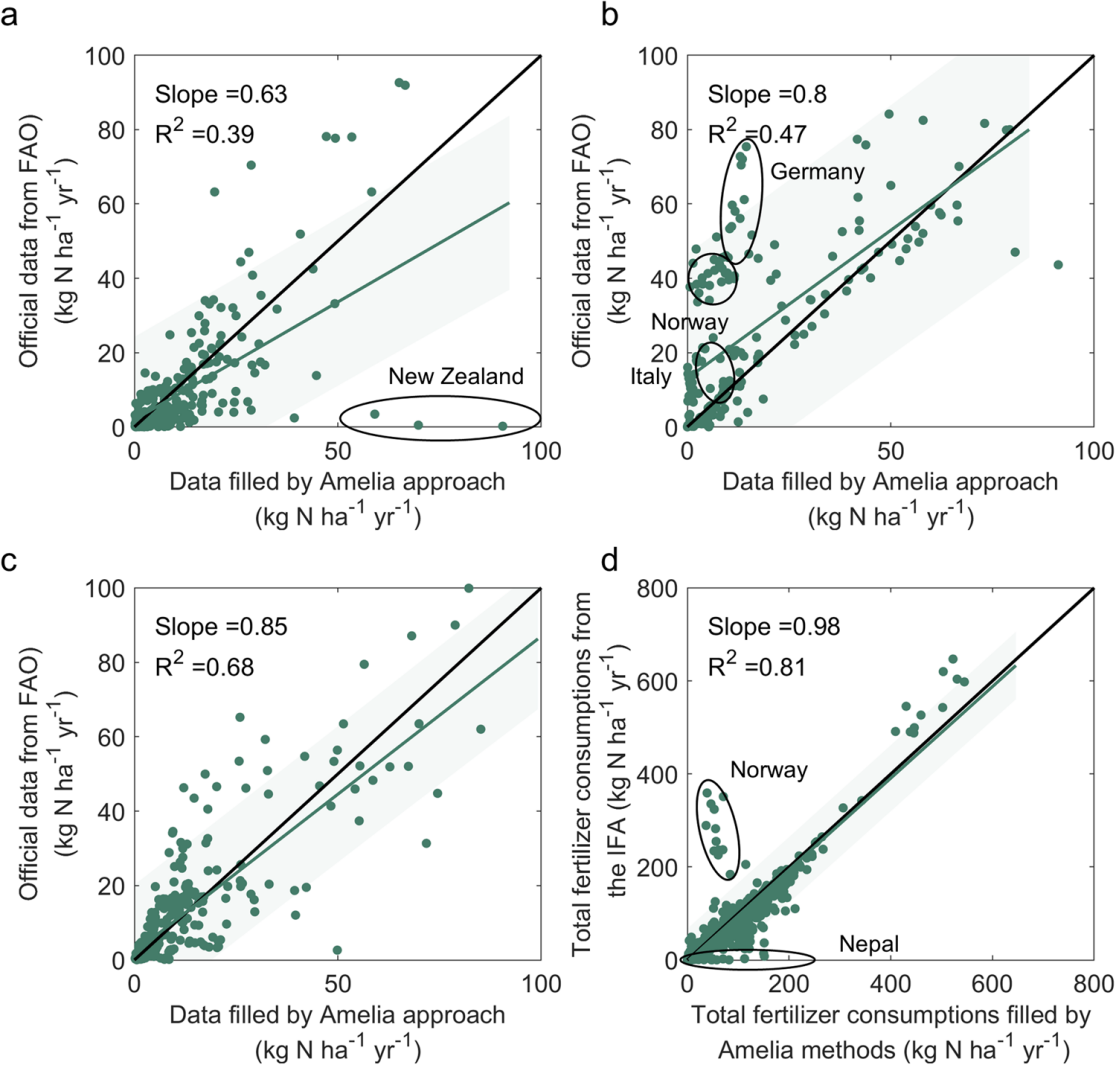
Comparison of country-level consumption per harvested area (kg N ha^−1^) of different fertilizer types during 1962–1972. Panel (**a**–**c**) represent comparison per ha of harvested area of urea, ammonium nitrate and ammonium sulphate filled by Amelia approach against official data from the FAOSTAT^20^, respectively. Panel (**d**) represents comparisons of sum of fertilizer consumption per ha of harvested area filled by Amelia approach against total fertilizer consumption from the IFASTAT^21^. Each point represents the fertilizer consumption of a specific country in a particular year.

In addition, we verified the reliability of the Amelia methods in the imputation of missing data by using national N fertilizer use by different types from the FAOSTAT^20^. Though, there were missing data from the FAOSTAT^20^, we still found 858 records of 69 countries during 1962–1972 overlapped the data filled by the Amelia approach. We directly compared the consumption of urea, ammonium nitrate and ammonium sulphate filled by Amelia method with those records from the FAOSTAT^20^. The scatter plots showed that the consumptions of these three N fertilizers per harvested area (kg N ha^−1^) were relatively close to the FAOSTAT^20^ (Fig. 10). However, there are also some estimates that are not accurate, such as urea for New Zealand, ammonium nitrate for Germany, Italy and Norway. Although the consumption of different N fertilizer types is missing for a few years from the IFASTAT^21^, the IFASTAT^21^ provides the total N fertilizer consumption amount for these years. Thus, we also calculated the summed-up N fertilizer consumption rate of different N fertilizer types filled by the Amelia methods and directly compared it with the IFASTAT^21^. It was confirmed that the total N fertilizer consumption rate filled by Amelia method in this study was very close to that from the IFASTAT^21^, except for Norway and Nepal.

**Fig. 11:**
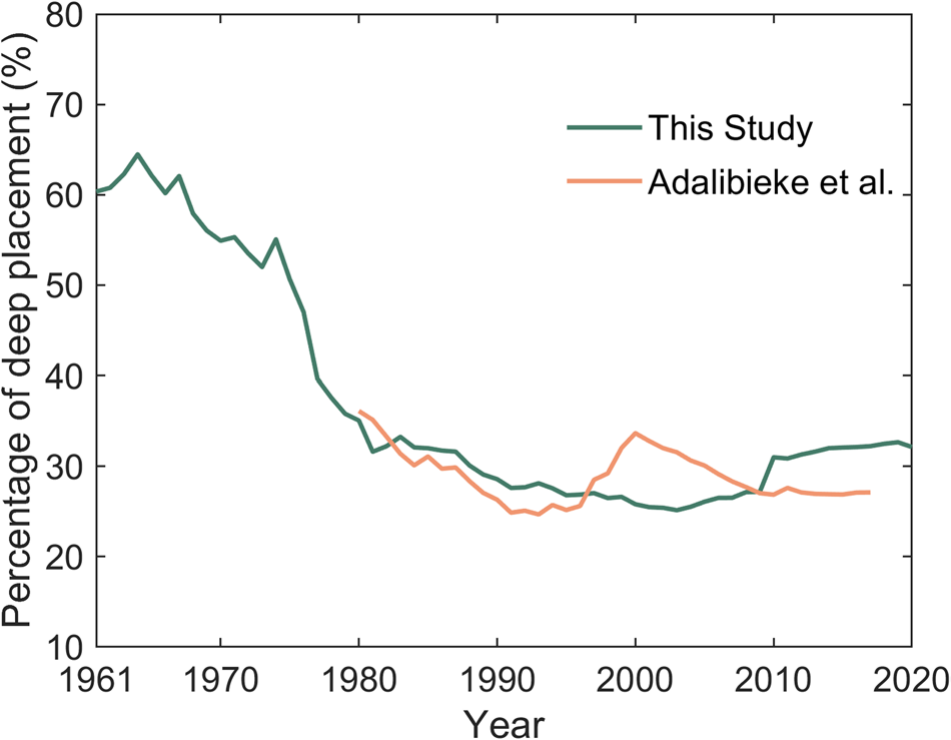
Comparison for the percentage of deep placement in China during 1980–2017 between this study and Adalibieke *et al*.^42^.

Lastly, although the data about fertilization placement was scarce, the comparison for regional scale (i.e., China) demonstrated that the values obtained by our estimation methods were consistent with that obtained by Adalibieke *et al*.^42^ (Fig. 11). It was noteworthy that the temporal trends about the percentage of N fertilization with the deep placement were close between these two studies, except in the year after 2000. Possible explanation is that the calculation of fertilization placement related to tillage fraction and fertilizer type in this study ignored some specific issues (e.g., the implementation of “Agricultural Cost-saving and Efficiency-increasing Program”, which encouraged the implement of deep fertilization machine to enhance fertilizer use efficiency and save agricultural cost for field crops), while the survey data can reflect more about the reality.

Overall, despite the inevitable uncertainties in our dataset, the analysis indicated that our data was aligned reasonably well with other existing multiple data sources or estimates provided by previous studies (including the FUBC^33^ report, USDA^56^, NDRCC^57,58^, FAOSTAT^20^ and so on). Needless to say, uncertainties related to this dataset can be easily reduced in the future as new underlying datasets become available or existing datasets are updated.

## Usage Notes

Our dataset provides a gridded estimate of the global crop-specific N application by fertilizer types and placements during the period of 1961–2020. Moreover, N application amount of each crop groups, NH_4_^+^ and NO_3_^−^ application can also be obtained from this dataset. For example, N application amount could be calculated by N application rate multiplied by harvested area. NH_4_^+^ and NO_3_^−^ application in synthetic N fertilizer could be calculated by N application of each fertilizer multiplied by composition of N types (i.e., NH_4_^+^ or NO_3_^−^).

However, there were several limitations that users should be aware when using this dataset. First, the historical cropland spatial and temporal distribution patterns provided by the HYDE3.2^31^ are inconsistent with the one from satellite-derived land use (e.g. China and India^59,60^) or national census (e.g. USA^61^) at regional scale. Therefore, the downscaling of crop-specific harvested area from cropland area might be biased, so did the calculation of the N application amount. Second, no significant interannual difference was assumed to the relative proportion of N application rate by each crop. The crop-specific N application rate was determined by the spatial patterns of N application amount over the study period and the relative proportion among crops, which is against the reality and survey data from FUBC^33^. We acknowledged the existence of this assumption and assessed the uncertainty quantitatively, however, we believe that uncertainty associated with this assumption will be reduced as new relevant data available. Third, this study aimed to provide a dataset about N fertilization practices by crop, however, we cannot obtain the data for the proportion of different types of N fertilizers applied by crop for such a long-term period from the current statistic data. Fourth, due to the lack of mechanical sowing and fertilization data, we assumed that fertilizers in non-tillage regions with high-income were deeply applied through fertilizer spreaders. While in reality, mechanical sowing and fertilization is more achievable than the conservation tillage (i.e., non-tillage), which means that cropland with tillage practice may be equipped with fertilizer spreaders and thus the percentages of N fertilization with deep placement are underestimated^40^. In addition, in our study, the non-tillage area was downscaled by crop type and the relationship between field size and non-tillage practices adoption, whereas the temporal coverage of field size was only available in the year 2015, assuming it constant during 1961–2020^41^. Thus, the non-tillage area in our study could be underestimated, since the field size has been increasingly associated with the economic growth and mechanization level^62^. Furthermore, the mechanical fertilization with deep placement in this study was also potentially underestimated. Furthermore, based on the analysis of uncertainties, the use of the N application rate for other cereals, oil crops, and rest of crops should be cautious, as well as the N application rate before the year 2000, the consumption of urea for New Zealand, and ammonium nitrate for Germany, Italy, and Norway. Lastly, although not quantified in this paper, we must acknowledge that other uncertainties arise from the use of multiple data sources and methodological choices, which can be considered in future studies.

Nevertheless, our study extended the crop-specific N fertilization for a long period of time and also provided a comprehensive dataset about N fertilization including N application rate, N fertilizer types and N fertilization placement simultaneously at uniform spatiotemporal resolution. In addition, we revealed a uniform approach using public and available datasets and methodologies to reconstruct N fertilization dataset over a relative long period of time. Such dataset can also be easily further updated when new underlying datasets and the update of existing datasets become available in the future.

Future directions of this study could focus on the conducting of finer scale and crop-specific surveys and reporting data on agricultural N fertilization practices. For example, the proportions of N fertilizers applied with different types varied by crops and geospatial distribution. The current survey data on different types of N fertilizer consumption from the IFASTAT^21^ and FAOSTAT^20^ are available at country-level and without dividing by detailed crop type. In comparison, the IFASTAT^21^ provided years of country-level N consumption data by crop types but not by fertilizer types. Therefore, continuous survey data of crop-specific N fertilizer consumption with different types at sub-national scales are necessary and critical for further improvement of our dataset. Moreover, application frequency and application timing are also crucial parts of agricultural N fertilization practices, suggesting that including the development of fertilizer application frequency and timing datasets could also be beneficial to improve our dataset. Last but not least, we can take advantage of satellite data and use machine learning and/or other comprehensive data fusion methods to improve the accuracy and precision of agricultural N fertilization practices^63^.

## Data Availability

Codes used in MATLAB 2020a and RStudio to deal with the dataset and plot the figures are available at available at National Tibetan Plateau/Third Pole Environment Data Center^55^.
